# Mycoplasma-Induced Rash and Mucositis (MIRM) mimicking herpetic stomatitis^☆^

**DOI:** 10.1016/j.abd.2025.501138

**Published:** 2025-06-28

**Authors:** Sara Becerril-Andrés, Adolfo Alejandro Cabanillas-Cabral, Gloria Baeza-Hernández

**Affiliations:** a Dermatology Department, Hospital Universitario de La Plana, Castellón, Spain; b Dermatology Department, Complejo Asistencial Universitario de Salamanca, Salamanca, Spain

Dear Editor,

A 20-year-old male presented with a 3-day history of painful oral lesions, impeding oral intake, and scrotal lesions. He recalled an episode of sore throat and cough 10 days prior, which had completely resolved after self-treatment with ibuprofen and two doses of amoxicillin-clavulanic acid. The patient had a history of herpes labialis but had not experienced an outbreak in recent months. He was otherwise well, afebrile, and without systemic symptoms.

On examination, confluent whitish aphthous lesions in a herpetiform pattern on the oral mucosa and mild gingivitis were noted (Fig. 1A‒D); along with three annular plaques with crusted center on the scrotal skin (Fig. 1E). There was no ocular or genital mucosal involvement. Cardiopulmonary auscultation and blood test, including complete blood count and general biochemistry panel, were normal. Microbiological samples and a scrotal lesion biopsy were collected.

**Figure 1:**
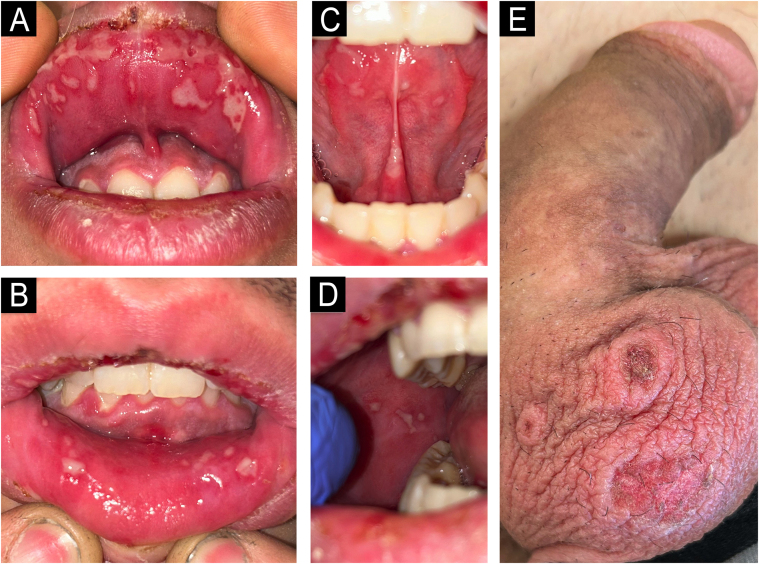
**Clinical images.** Confluent whitish aphthous lesions in a herpetiform pattern on the oral mucosa and mild gingivitis (A‒D). Three annular plaques with crusted center on the scrotal skin (E).

The initial differential diagnosis included Mycoplasma-Induced Rash and Mucositis (MIRM) or Reactive Infectious Mucocutaneous Eruption (RIME); toxicodermia within the Erythema Multiforme (EM) ‒ Stevens-Johnson Syndrome (SJS) ‒ Toxic Epidermolytic Necrosis (TEN) spectrum; and herpetic stomatitis with minor EM. The patient was initiated on dexamethasone 4 mg daily for three days, valacyclovir, and topical triamcinolone acetonide. Upon follow-up 72 hours later, the lesions had disappeared.

Serology was positive for Mycoplasma Pneumoniae (MP) (both IgM and IgG antibodies), Herpes Simplex Virus (HSV) (IgG antibodies only), and negative for HIV, hepatitis viruses, and syphilis. Polymerase Chain Reaction (PCR) from oral lesions was negative for HSV types 1 and 2.

The biopsy revealed a dense inflammatory infiltrate in the superficial and mid-dermis, predominantly composed of lymphocytes and polymorphonuclear cells. Interface damage was evident, with subepidermal splitting and apoptotic keratinocytes (Fig. 2).

**Figure 2:**
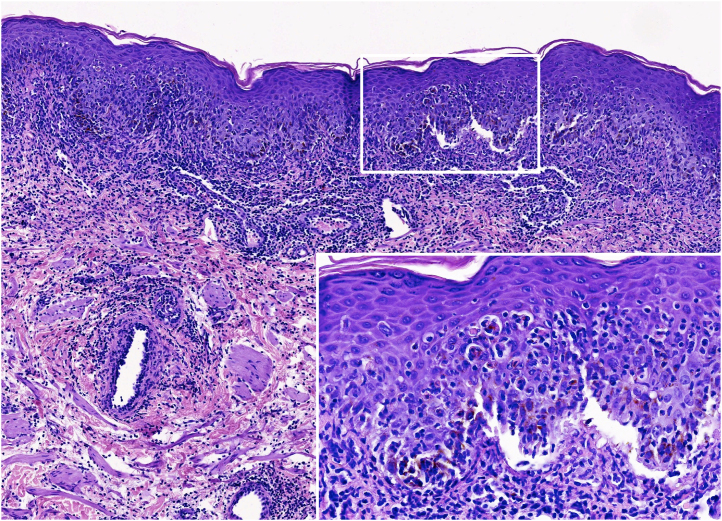
**Histological images.** Scrotal lesion showing a dense inflammatory infiltrate in the superficial and mid-dermis, predominantly composed of lymphocytes and polymorphonuclear cells. Interface damage is evident, with subepidermal splitting and apoptotic keratinocytes (Hematoxylin & eosin; ×160 and ×800 – inset).

Acute mucositis with minimal or absent skin involvement has been described under terms like “atypical SJS”, “SJS without skin lesions”, and “Fuchs syndrome”.^1^ In 2015, Canavan et al. introduced the term MIRM to distinguish the unique mucocutaneous eruptions linked to MP from drug-induced SJS, TEN, and herpes-related EM.^1^, ^2^, ^3^, ^4^ In recent years, other pathogens such as Chlamydophila Pneumoniae (CP), influenza B, parainfluenzavirus, adenovirus, metapneumovirus, rhinovirus, enterovirus, group A *Streptococcus*, and SARS-CoV-2 have been reported to trigger similar mucocutaneous reactions.^1^, ^2^ This has led to the concept of RIME, which reflects the spectrum of infectious agents capable of inducing these eruptions.^1^, ^2^

MIRM is an uncommon condition, predominantly affecting children and young adults, marked by prominent mucositis with limited skin involvement, typically preceded by non-specific flu-like symptoms that manifest 7‒10 days before mucocutaneous onset.^1^ The vesiculobullous and atypical targetoid skin lesions bear a resemblance to those in EM, SJS, and TEN. However, MIRM presents with distinctive clinical, pathophysiological, and prognostic outcomes that set it apart from these conditions.^2^, ^3^, ^4^ The differential diagnosis should also encompass viral infections (e.g., HSV, Epstein-Barr, cytomegalovirus, Coxsackie A6, and HIV), oral candidiasis, exposure to caustic substances, and autoimmune diseases like pemphigus vulgaris.^4^, ^5^, ^6^

The pathophysiology underlying MIRM remains unclear; however, it is hypothesized to involve polyclonal B-cell proliferation and antibody production following MP infection, leading to immune complex deposition and complement activation. Additionally, molecular mimicry between mycoplasma P1 adhesion molecules and a keratinocyte antigen, as well as genetic susceptibility, have been proposed.^4^

Histologically, MIRM/RIME lesions share features with EM, SJS, and TEN, including apoptotic keratinocytes, full-thickness epidermal necrosis with subepidermal splitting, and superficial dermal infiltrate with sparse perivascular lymphocytes.^1^, ^5^, ^6^

PCR has emerged as the “gold standard” for establishing the microbiologic etiology of community-acquired pneumonia, offering higher sensitivity to detect MP or CP, particularly in the earlier stages of infection.^5^, ^7^ Specific serological detection can be valuable for retrospective diagnosis, particularly when samples are taken at least two weeks apart to assess seroconversion or a fourfold increase in antibody titers.^4^, ^5^, ^7^ MP-IgM antibodies appear within one week of clinical onset, peaking around the third week, and serving as a marker of recent infection.^5^, ^7^ MP-IgG antibodies appear about two weeks post-infection, peak at five weeks, and persist long-term.^5^, ^7^

The prognosis is generally good, with low rates of sequelae and a good therapeutic response to antibiotics and/or systemic steroids.^4^

This case highlights the importance of considering MIRM/RIME in the differential diagnosis of mucositis. The history of recent respiratory symptoms without drug exposure may suggest MP or CP infection, helping to distinguish these conditions from SJS and TEN. Early recognition and targeted treatment are essential for optimal management.

## Authors' contributions

Sara Becerril Andrés: Had access to the data and played a role in writing this manuscript.

Adolfo Alejandro Cabanillas Cabral: Had access to the data and played a role in writing this manuscript.

Gloria Baeza-Hernández: Had access to the data and played a role in writing this manuscript.

## Financial support

None declared.

## Conflicts of interest

None declared.
